# Cystic Lung Disease Presenting as Recurrent Non-traumatic Chylothorax: Case Report and a Mini-Review

**DOI:** 10.7759/cureus.40217

**Published:** 2023-06-10

**Authors:** Sushan Gupta, Vishesh Paul

**Affiliations:** 1 Internal Medicine, Carle Foundation Hospital, Urbana, USA; 2 Pulmonology and Critical Care, Carle Foundation Hospital, Urbana, USA

**Keywords:** pleural effusion, recurrent chylothorax, chylothorax, sporadic lymphangioleiomyomatosis, cystic lung disease

## Abstract

Malignancy and infections are the most common causes of recurrent chylothorax. Cystic lung disease, especially sporadic pulmonary lymphangioleiomyomatosis (LAM), is a rare condition that may manifest as recurrent chylothorax. We present a case of a 42-year female who presented with dyspnea on exertion secondary to recurrent chylothorax, requiring three thoracenteses within a few weeks. Chest imaging showed multiple bilateral thin-walled cysts. Thoracentesis revealed milky-colored pleural fluid, which was exudative and lymphocytic predominant. Infectious, autoimmune, and malignancy workup was negative. Vascular endothelial growth factor-D (VEGF-D) levels were sent for testing, which came back elevated (2001 pg/ml). A presumptive diagnosis of LAM was made based on recurrent chylothorax, bilateral thin-walled cysts, and elevated VEGF-D levels in a reproductive age group woman. Given quick reaccumulation of chylothorax, she was started on sirolimus. After initiating therapy, there was a significant improvement in the patient's symptoms, with no recurrence of chylothorax in the five years of follow-up. Awareness of different forms of cystic lung diseases is vital to establish an early diagnosis, which may prevent disease progression. Rarity and heterogeneity of presentation often make the diagnosis challenging, requiring a high degree of suspicion.

## Introduction

Chylothorax, or collection of chyle in the pleural fluid, is a relatively uncommon cause of pleural effusion in adults. Obstruction of trans-diaphragmatic chyle flow in the thoracic duct or its tributaries leads to extravasation into the pleural cavity [1]. Identifying the underlying etiology, especially in patients with recurrent chylothorax, is vital due to the associated morbidity and mortality. Malignancy and infection are often the primary differentials among patients with non-traumatic recurrent chylothorax [2]. However, diagnosing the cause becomes exponentially challenging once they are excluded. We present a case of a middle-aged woman with recurrent chylothorax and diffuse cystic lung disease, identified as pulmonary lymphangioleiomyomatosis (LAM).

## Case presentation

Our patient was a 42-year-old woman who presented to the pulmonary clinic with progressive shortness of breath on exertion. A review of symptoms was negative for fever, cough, hemoptysis, frequent respiratory tract infections, weight gain, orthopnea, unilateral leg swellings, or any neurological symptoms. Her only known medical history was mild intermittent asthma, rarely requiring albuterol. She had two children and worked as a social worker. She had no significant smoking history and had been on hormone replacement therapy for dysfunctional uterine bleeding until two years back. Her family history was significant for hypertension and cardiac disease but no chronic lung or neurological problems. Her travel history was non-contributory.

**Figure 1:**
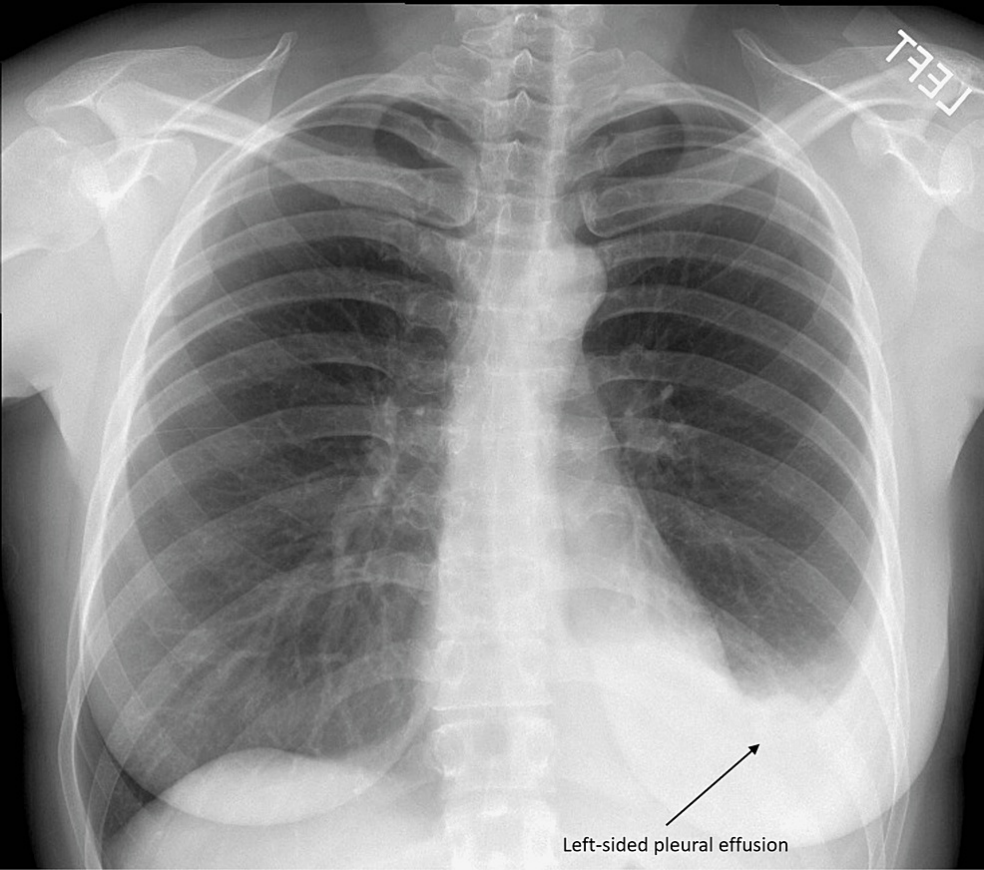
Chest X-ray demonstrating left-sided pleural effusion.

On the exam, she had stable vital signs, and her SpO2 was 95% on room air. Lung auscultation revealed diminished breath sounds at the left lung base, and the rest of the exam was within normal limits. A chest X-ray showed a left-sided pleural effusion (Figure 1).

**Figure 2:**
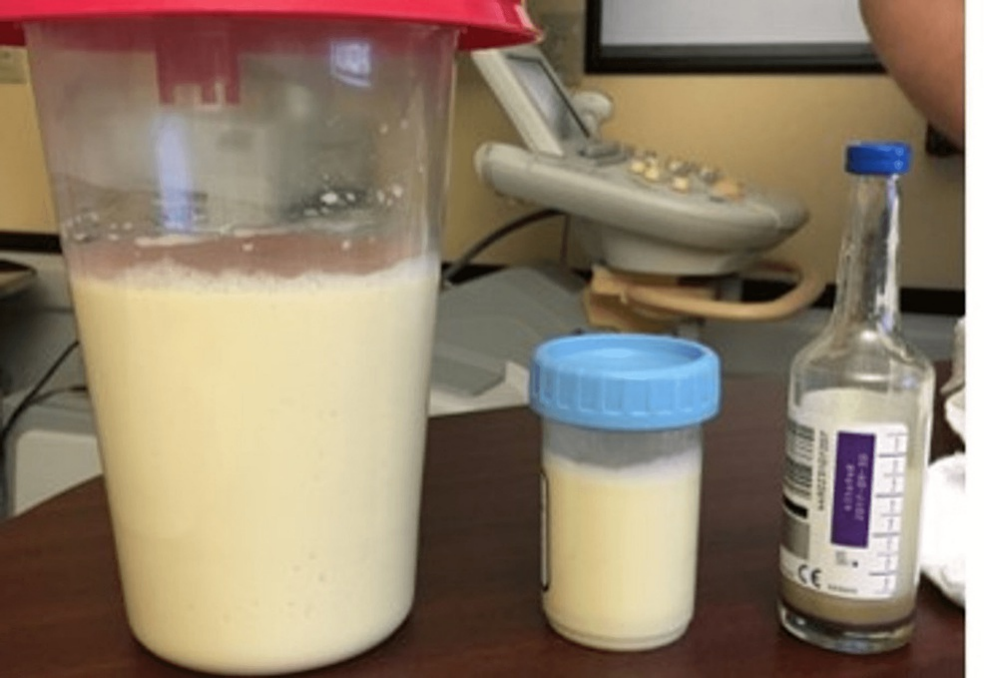
Chylous fluid removed after thoracentesis.

Thoracentesis was performed, draining 1000 mL of chylous-looking fluid (Figure 2).

**Figure 3:**
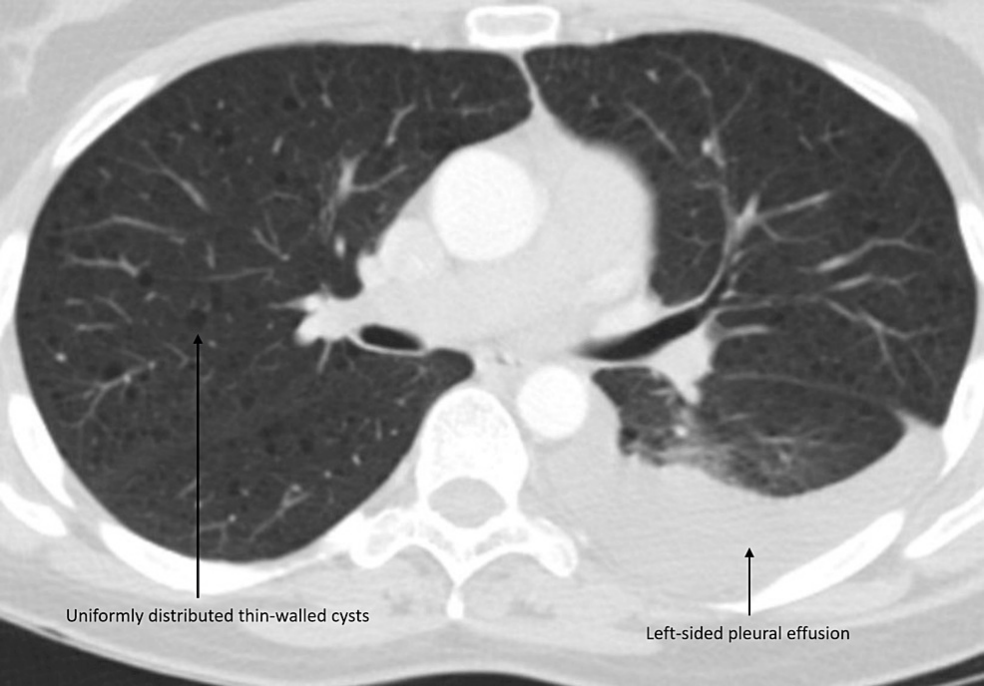
CT chest demonstrating diffusely distributed thin-walled cysts in both lungs with moderate left pleural effusion.

Pleural fluid testing revealed the following: lymphocytic predominance (68%), lactate dehydrogenase (LDH) 117 U/L, protein 4.6 gm/dL (serum protein 6.7gm/dL), triglyceride level (2363 mg/dL), cholesterol less than 50 mg/dL, and glucose 76 mg/dL. Pleural fluid cytology showed reactive mesothelial cells and abundant small lymphocytes with no malignant cells. Infectious workup, including bacterial, fungal, and mycobacterial cultures, showed no growth. Her pleural fluid reaccumulated rapidly, requiring thoracentesis thrice within a few weeks. A computed tomography scan (CT scan) of the chest showed multiple thin-walled cysts throughout the lungs without sparing costophrenic angles (Figure 3).

A PET-CT scan was also done to rule out any malignancy as a cause for the chylothorax and did not reveal any area of abnormal uptake.

Alpha-1 antitrypsin and anti-Sjögren's-syndrome-related antibody (SSA/SSB) levels were sent for the workup of cystic lung disease and were normal. Vascular endothelial growth factor - D (VEGF-D) level came back elevated at 2001 pg/ml (normal <600 pg/mL). Given typical findings of bilateral thin-walled cysts in a nonsmoking female of reproductive age group and recurrent chylothorax, a diagnosis of LAM was established. Based on the above-mentioned vital findings, a surgical lung biopsy was considered unnecessary and was avoided. Her pulmonary function test did not reveal any obstructive or restrictive pathology.

Given frequent reaccumulation of pleural fluid, she was started on a mechanistic target of rapamycin (mTOR) inhibitor, sirolimus 1 mg daily. There was a significant improvement in the patient's symptoms after initiating therapy. She has remained active with no reaccumulation of pleural fluid in five years of follow-up since initiating therapy. There was a slight decline in forced vital capacity (FVC) on follow-up pulmonary function tests (PFTs), with a slight increase in the number and size of cysts on follow-up lung CT scans. However, as she has remained asymptomatic, the same dose of sirolimus has been continued with regular monitoring of her PFTs.

## Discussion

The thoracic duct originates from the cisterna chyli at the level of the second vertebra and carries approximately two liters of chyle daily [1]. Chylothorax represents a lymphocyte-predominant exudative pleural effusion with a classical milky appearance [1]. Pseudo-chylothorax, which is seen in tuberculosis and rheumatoid disease and mimics the milky appearance of chylothorax, is often excluded based on a lower triglyceride (<110 mg/dL) and higher cholesterol (>200 mg/dL) content [3]. Chylothorax is commonly unilateral, with the right side being more common than the left [1]. 

Iatrogenic or traumatic chyle leaks are seen in approximately 50% of cases of chylothorax. Non-traumatic chylothoraces, on the other hand, need an extensive workup to establish the cause, with lymphoproliferative malignancies constituting the majority of them [2]. Other malignancies, such as lung cancer, leukemias, mediastinal tumors, Kaposi sarcoma, and myeloma, have also been associated with chylothorax [2]. Infectious causes like tuberculosis and filariasis, especially in tropical countries, constitute other common causes [2].

LAM is a rare cause of recurrent chylothorax in women of reproductive age, with an incidence of 3.4-7.8/1,000,000 in women worldwide [4]. LAM cells are immature smooth muscle cells in the bloodstream and lymph glands. Activated mTOR pathways stimulate the proliferation of LAM cells which express VEGF, allowing metastasis and angiogenesis into the lung tissue, leading to lung parenchymal destruction and cyst formation [5,6]. The commoner subtype is associated with tuberous sclerosis, and a careful clinical exam is needed to identify signs of cutaneous TSC [7]. Sporadic LAM is extremely rare, with an estimated prevalence of 2-4 per million, and seen almost exclusively in women [8]. It is usually diagnosed between the third and fourth decades of life [7]. LAM cells express estrogen and progesterone receptors which might be responsible for female predominance and decline in lung function with estrogen [8]. The typical presentation of LAM includes symptoms of progressive dyspnea on exertion and sometimes spontaneous pneumothorax. In approximately 10-30% of cases, recurrent pleural effusion (chylothorax) may be the reason that makes the person seek medical care [7]. Pulmonary function testing is initially normal. As the disease progresses, it often shows airflow obstruction similar to chronic obstructive pulmonary disease (COPD) and asthma, making it easy to misdiagnose LAM without advanced lung imaging [9]. This is especially true for COPD, as it is easy to confuse emphysema with cysts on lung CT, and PFTs in both diseases would show an obstructive pattern.

Cystic lung disease represents a group of uncommon lung diseases with varied clinical presentations. Identifying the underlying etiology is often a diagnostic challenge, and understanding the differences in their pathophysiology and imaging pattern on CT scans is essential to establish the diagnosis. Some of the known conditions associated with cystic lung disease include LAM, pulmonary Langerhans cell histiocytosis (LCH), lymphocytic interstitial pneumonia (LIP), Birt-Hogg-Dube syndrome (BHD), amyloidosis, and light-chain disease (LCD).

LCH, universally associated with smoking, is a nodular lung disease characterized by irregularly shaped diffuse centrilobular and peribronchiolar nodules caused by the infiltration of Langerhans cells into the lung parenchyma. Some of these nodules in LCH may cavitate as the disease progresses to form cysts and present as nodulocystic lesions. A classic finding in LCH is the relative sparing of the costophrenic angles [10].

LIP is caused by lymphocytic infiltration of the alveoli and interlobular septa, leading to the formation of lymphoid aggregates [11]. CT scan typically shows irregular nodules, ground-glass opacities, focal consolidations, and peri broncho vascular thickening [12]. More than 70% of cases have cysts on the scan. LIP is usually associated with connective tissue disorders (such as systemic lupus erythematosus and Sjogren syndrome) and HIV; hence, serological testing (SSA, SSB, HIV antibody) is warranted [6].

Amyloidosis is a rare systemic disorder that can also present as isolated pulmonary involvement with parenchymal infiltrates, nodules, pleural disease, and peripherally distributed cysts [13]. LCD, similar to amyloidosis, is characterized by the deposition of immunoglobulin light chains in the lung parenchyma. However, these light chains do not form amyloids and are differentiated based on the negative Congo-red stain [10]. LCD is predominantly associated with myeloma, and the CT scan usually shows diffusely distributed nodules and cysts [6,14]. 

The two primary differentials for cystic lung disease in the absence of associated lung nodules and normal intervening lung parenchyma include BHD and LAM. BHD, aka folliculin gene-associated syndrome, is an autosomal dominant dermatological disorder characterized by fibrofolliculomas, trichodiscomas over the face, and diffusely distributed skin tags [15]. Pulmonary cysts in BHD are elliptical and lentiform-shaped, often subpleural, and distributed predominantly in the lower lung zones [6,10]. Emphysema due to alpha-1 antitrypsin deficiency and infectious causes like *Pneumocystis carinii* can also mimic the appearance of cystic lung diseases, and the medical team should be mindful of them too.

The reproductive age group and characteristic imaging findings of thin-walled uniform cysts diffusely distributed in both lungs with normal adjacent lung parenchyma strongly suggested our patient's diagnosis of LAM [16]. Lung biopsy is the gold standard for confirming the diagnosis; however, elevated VEGF-D levels, especially > 800 pg/ml in our patient, were diagnostic, avoiding lung biopsy [17]. Serum VEGF-D levels are elevated in up to 70% of cases of LAM [18]. The management of LAM has undergone a paradigm change after the MILES trial, which showed stability in lung function with sirolimus in patients with LAM compared to the progressive 10% decline seen with placebo [19]. Sirolimus is an immunosuppressant agent that acts on the mTOR pathway inhibiting the proliferation and migration of the LAM cells [20]. These medications are not curative but stabilize lung function and improve symptoms. Periodic blood testing is done to maintain serum sirolimus levels between 5-15 ng/ml [19]. Our patient has done significantly well after initiating sirolimus with no recurrence of chylothorax in the five years of her follow-up. 

## Conclusions

LAM is a rare cause of non-traumatic recurrent chylothorax. Diffusely distributed thin-walled lung cysts with elevated VEGF-D levels in a reproductive age group women are diagnostic and preclude the need for a surgical lung biopsy. Medical providers must be aware of the spectrum of cystic lung diseases (and how they can be commonly misdiagnosed as emphysema) and the required diagnostic work to establish an early diagnosis to prevent disease progression.
